# The Impact of Vitamin D Low Doses on Its Serum Level and Cytokine Profile in Multiple Sclerosis Patients

**DOI:** 10.3390/jcm10132781

**Published:** 2021-06-24

**Authors:** Anna Walawska-Hrycek, Weronika Galus, Eugeniusz Hrycek, Aleksandra Kaczmarczyk, Ewa Krzystanek

**Affiliations:** 1 Department of Neurology, Faculty of Medical Sciences in Katowice, Medical University of Silesia, 40-752 Katowice, Poland; awalawska-hrycek@sum.edu.pl (A.W.-H.); wgalus@sum.edu.pl (W.G.); akaczmarczyk@sum.edu.pl (A.K.); 2 Department of Cardiology, Faculty of Medical Sciences, Andrzej Frycz Modrzewski Krakow University, 30-705 Krakow, Poland; ehrycek@gmail.com

**Keywords:** vitamin D, supplementation, cytokine, multiple sclerosis

## Abstract

Vitamin D is known to have immunomodulatory properties and its deficiency is identified as an environmental risk factor for the development of autoimmune diseases, including multiple sclerosis. The aim of this study was to assess whether low-dose vitamin D supplementation could normalize the 25(OH)D serum levels in patients with relapsing-remitting multiple sclerosis (RRMS) and vitamin D deficiency (serum 25(OH)D < 75 nmol/L), and whether it may impact serum levels of selected cytokines. Among 44 patients (mean age 38.4 ± 10.1 years, 33 women and 11 men), after 12 months of low-dose vitamin D supplementation, serum levels of 25(OH)D normalized in 34 (77.3%) of the patients. Together with vitamin D increase, median levels of anti-inflammatory cytokines (IL10, TGF-β) and regulatory IFN-γ increased, while proinflammatory IL-17 remained unchanged. Moreover, an increase of inorganic phosphorus levels and decrease of PTH levels were observed, but without changes in total calcium concentration. These results may indicate that long-term supplementation with low doses of vitamin D is sufficient to compensate its deficiency in patients with RRMS and may help to maintain beneficial anti-inflammatory cytokine profile.

## 1. Introduction

Vitamin D is a family of compounds that is essential for the proper growth and formation of teeth and bones and to help regulate the immune system [1,2]. The family includes: Ergocalciferol (Vitamin D2), Cholecalciferol (Vitamin D3), Calcifediol (25-hydroxyvitamin D, 25(OH)D3) and Calcitriol (1,25 dihydroxy-vitamin D, 1,25(OH)2D3). Vitamin D deficiency is a well-known environmental risk factor for the development of autoimmune diseases, including multiple sclerosis (MS) [3].

Vitamin D comes from two sources: endogenous, which is produced in the skin un-der exposure to sunlight, and exogenous, which is ingested in foods and supplements. Inactive vitamin D precursors under the double hydroxylation in the liver and kidneys are transformed into active metabolite calcitriol [3]. Higher risk of deficiency occurs in elderly or obese people, in people with low exposure to direct sunlight, people with darker skin, and people who take certain medications for long periods of time.

Vitamin D receptors (VDR) are found on the bone cells (osteoblasts, chondrocytes), in the parathyroid and pituitary glands, gastrointestinal tract, kidneys, lungs and many other locations. They are also found on cells and tissues of the immune system (e.g., thymus and spleen), which may explain the immunomodulatory effects of calcitriol [4]. One of the most important are dendritic cells (DC), which belong to the antigen-presenting cells (APC), generating an immune response and inducing immunological tolerance [5]. In the presence of calcitriol, DC change their phenotype into tolerogenic DC by lowering the levels of major histocompatibility II complex (MHC II) and costimulatory molecules [6]. Tolerogenic DC secrete anti-inflammatory cytokines, induce T-cell anergy and inhibit the migration of Th1 and Th17 cells from peripheral organs into the CNS [6]. Vitamin D modulates lymphocytes T and promotes switch from Th1 into anti-inflammatory Th2 phenotype, it also inhibits T cells differentiation into Th17 [7]. All these actions of calcitriol lead to reduced production of proinflammatory cytokines [8].

There are several cytokines identified to play important role in the neuroinflammation process in MS, which are modulated by the vitamin D metabolism:

- IL-10 acts as immunosuppressive mainly on regulatory T cells, macrophages and inhibits the MHC II in DC [9,10]. It can also stimulate the immune response to enhance B cell survival and antibody production [11]. The biological effect of IL-10 also applies to neuroprotective processes in both neuron and glial tissues [12]. This is important due to the neurodegenerative processes following the inflammatory phase in the course of MS [12]. A transcription of IL-10 is regulated by 1,25(OH)2D3 [13].
- Transforming growth factor β (TGF-β) is a regulatory cytokine, which determines the type of response and modifies immune function [14]. TGF-β reduces neurological damage and CNS lesions [15].
- Interleukin 17 (IL-17) is the main proinflammatory cytokine released by activated lymphocytes Th17 [16,17]. Due to its ability to cross the blood–brain barrier and develop demyelination in the CNS, IL-17 is as a major contributor of MS immunopathogenesis [16,18]. Increased IL-17 level has also been detected in MS lesions and cerebrospinal fluid [18,19,20]. The differentiation of Th17 can be inhibited in the presence of calcitriol via VDR receptor [1,16,17].
- Interferon gamma (IFN-γ) is a proinflammatory lymphokine that plays an important role in the innate and adaptive immune system by modulating cellular processes [21]. IFN-γ action is implemented by regulation of MHC I and II, differentiation of T cells to a Th1 phenotype, and activation of B cells to induce immunoglobulin secretion [21,22]. A proinflammatory IFN-γ activity can be inhibited by vitamin D [23]. 1,25(OH)2D3 decreases the synthesis of INF-γ in peripheral blood lymphocytes (PBL) and T cell lines [23].

Although vitamin D supplementation is quite frequent in the routine clinical practice in patients with MS, especially in case of deficiency, there are still no clear guidelines on vitamin D supplementation addressing both bones’ health and the impact of vitamin D on the immunological system [3,16,24,25]. Available dosing schemes differ substantially between studies (800–10,000 IU/day) ([18,20]), thus it is difficult to determine the benefit of supporting proper a 25(OH)D concentration in patients with MS, which may result in maintaining of adequate calcium-phosphate balance and favorable modulation of anti-inflammatory mediators. Therefore, the aim of this study was to assess whether low-dose vitamin D supplementation could normalize the 25(OH)D serum levels in patients with relapsing-remitting multiple sclerosis (RRMS) and vitamin D deficiency, and whether it may impact selected cytokines’ serum levels.

## 2. Materials and Methods

### 2.1. Study Group

Eighty-three patients with RRMS diagnosed according the 2010 revised McDonald criteria [26], who were treated in the Department of Neurology and Neurology Outpatient Clinic of the Medical University of Silesia in Katowice, Poland, were selected during the winter season (from November to March) and invited to participate in the study.

Patients were recruited according to the following inclusion criteria:

- Aged between 18 and 55 years;
- Diagnosis of RRMS based on the McDonald Criteria (2010) [26];
- Stable MS disease modifying therapy for at least 6 months prior to the study;
- Vitamin D deficiency, as defined as 25(OH)D serum level below 75 nmol/L.

The exclusion criteria were as follows:

- Diagnosis of PPMS or SPMS;
- Renal or liver failure, thyroid dysfunction, hyperparathyroidism;
- Depression;
- Anemia, leucopenia;
- Epilepsy;
- Pregnancy, breast-feeding;
- Malabsorption or taking medication affecting calcium-phosphate metabolism within the last 6 months;
- Spending most time indoors, working underground, traveled to a different climate zone within the last 6 months;
- Potential dysfunction of the immune system (e.g., MS relapse within the last 30 days), steroid therapy taken within the last 30 days, overt symptoms of acute or chronic inflammation.

The study protocol was approved by the Bioethics Committee of Medical University of Silesia (approval no. KNW/0022/KB1/121/14). All participants provided written in-formed consent.

### 2.2. Methods

At baseline, with the use of standardized questionnaire, a basic information on patients’ demographics and selected lifestyle features was collected, e.g., average time spent outdoors under the sunlight, intake of vitamin D, possible dietary sources (oily sea fish, offal foods), or smoking. Baseline clinical characteristics (e.g., age at first symptoms appearance, disease duration, type of onset) were collected from the available clinical records.

Neurological examination, together with EDSS scoring (Expanded Disability Status Scale) [27], and laboratory blood assessments were performed twice during the study, at baseline and after 12 months. The 25(OH)D is the vitamin D metabolite usually measured in the blood since it is representative of the vitamin D stored in the organism. Levels of vitamin D, as measured by 25-hydroxyvitamin D (25(OH)D) levels, and selected cytokine levels (INF-γ, IL-17, IL-10, TGF-ß) were measured using ELISA (enzyme-linked immuno-sorbent assay) kits and spectrophotometer µQuant ELISA. To control the safety of long-term supplementation with vitamin D, we have controlled the parameters of calcium and phosphate metabolism: total blood calcium, inorganic phosphorus and PTH (parathyroid hormone).

All participants included to the study were administered oral vitamin D for the next 12 months at the dose depending on the degree of its deficiency. Vitamin D deficiency and dose of vitamin D related to its initial level was defined as shown in Table 1.

During the study period, disease modifying therapy was not changed, nor did the patients receive any additional medication.

On the final assessment, compliance was estimated according to the Kampman formula: compliance (%) = number of used vitamin D pills/number of administered vitamin D pills × 100%. Compliance between 80% and 120% was considered satisfactory.

### 2.3. Statistical Analysis

Statistical analysis was performed using the Statistica version 12.0 software (TIBCO Software Inc., Palo Alto, CA, USA). The Shapiro–Wilk test was used to assess distribution. Data were presented as mean ± standard deviation (SD), or median with minimal and maximal values, where applicable. The Wilcoxon signed-rank test was used for intragroup comparisons and Mann–Whitney U test was used for between-group comparisons. Changes in proportions of selected groups of vitamin D deficiency were assessed with McNemar test for symmetry. Spearman’s rank order correlation test was used to test the mutual correlations between the studied parameters. A *p* < 0.05 was considered statistically significant.

## 3. Results

### 3.1. Study Group

In 83 RRMS patients selected, 10 patients were previously treated with vitamin D and 3 had 25(OH)D levels above 75 nmol/L. During the study, 18 patients failed to show up for the second blood sampling (including 16 lost to follow-up and 2, who became pregnant) and 8 patients changed MS center (withdrawn their consent). Therefore, only the data of 44 patients (mean age ± SD 38.4 ± 10.1 years, 33 women, 75%) were included in the further analysis. All analyzed patients were receiving a Disease Modifying Treatment (DMT). The demographic and clinical characteristic of included patients is shown in Table 2.

### 3.2. Vitamin D Serum Level

At baseline, 31 (70.4%) patients had vitamin D deficiency (25(OH)D levels ≤50 nmol/L), thus were supplemented with 1000 IU vitamin D, and 13 (29.5%) had insufficiency (25(OH)D levels 50–<75 nmol/L), and therefore were supplemented with 500 IU vitamin D. After one year of vitamin D supplementation, levels of 25(OH)D were considered normal in 34 (77.3%) out of 44 patients (Table 3). However, among 31 patients with greater deficiency, who were supplemented with 1000 IU vitamin D, 23 (74.2%) patients achieved normal level, and among those with smaller deficits, who were treated with 500 IU vitamin D, 11 (84.6%) reached normal level of 25(OH)D.

The median serum concentration of 25(OH)D at baseline was 39.8 nmol/L, whereas at the end of study it significantly increased to 91.2 nmol/L (*p* < 0.001) (Table 4).

### 3.3. Cytokines Serum Levels

After 12 months of vitamin D supplementation, significant increase in serum concentrations of IL-10, TGF-β, IFN-γ but not IL-17 were observed. The levels of cytokines in MS patients are presented in the Table 4.

### 3.4. Parameters of Calcium-Phosphate Metabolism

After 12 months of vitamin D supplementation, an increase of inorganic phosphorus levels and decrease of PTH levels were observed, but without changes in total calcium concentration (Table 4).

### 3.5. Relationship between Vitamin D Concentration and Selected Cytokines Levels and Calcium-Phosphate Metabolism

After 12 months of vitamin D supplementation, no correlation between changes in IL-10, TGF-β, IL-17 and IFN-γ serum levels with overall increase of vitamin D level was found. PTH serum levels decrease was significantly inversely correlated with 25(OH)D levels increase (R = –0.32, *p* = 0.036), but there was no correlation regarding inorganic phosphorus and total calcium levels. At month 12, there was no significant difference in the observed changes in any of analyzed cytokines or calcium-phosphate metabolism parameters between patients who reached normal levels of 25(OH)D and those with existing vitamin D deficit.

### 3.6. Relationship between Vitamin D Concentration and EDSS Score

The median (min–max) EDSS score 1.5 (0–4) did not change during the study and was the same after one-year supplementation as at baseline; therefore, the relationship with 25(OH)D levels was not assessed.

### 3.7. Compliance

Satisfactory compliance of vitamin D administration was achieved in 39 (88%) patients.

## 4. Discussion

### 4.1. The Effect of Vitamin D Supplementation

The results of this study suggest that low-dose vitamin D supplementation (500 or 1000 IU daily) in patients with RRMS and vitamin D deficits may be sufficient to achieve normalization of 25(OH)D serum levels. In many randomized clinical trials assessing immunological or clinical effects of vitamin D, doses were much higher to achieve sufficient clinical or immunological response [17,18,19,20,29,30,31,32]. It seems yet to be determined whether the key of supplementation of vitamin D in patients with MS is to reach its normal levels or to activate immunological response. The doses for these two objectives may be different.

The effectiveness of vitamin D supplementation may be related to treatment compliance. Although, even in the setting of randomized clinical trials, 100% compliance is not achievable [33]. In our study, only 12% of patients confirmed noncompliance, which can be considered negligible for the final results.

### 4.2. The Effect of Vitamin D on the Concentration of the Inflammatory Cytokines

After 12 months of intervention, an increase of concentration of anti-inflammatory cytokines (IL-10, TGF-β) and a regulatory IFN-γ were observed, with stable levels of IL 17. Similar effects of immunological response to Vitamin D supplementation were reported in several previous studies; however, in majority of them this response was observed after higher doses than in our study. Selected previous reports are provided in Table 5.

#### 4.2.1. IL-10 Serum Levels

In the presented study, IL-10 concentration increased after 12 month of low-dose vitamin D supplementation. Similar results were achieved by Ashtari et al. and Farsani et al. [30,34]; however, they used much higher doses: 50,000 IU every 5 days or every week, respectively. Moreover, Farsani et al. reported 3.84 times higher expression for the IL-10 gene among MS patients supplemented with vitamin D [34].

#### 4.2.2. IL-17 Serum Levels

The active form of vitamin D affects lymphocytes that release IL-17 [18]. In our study, no changes in the concentration of IL-17 after 12 months were found. Sotirchos et al. re-ported that after using a large doses of vitamin D a decrease in the concentration of pro-inflammatory IL-17 was observed, which did not occur in the group taking lower doses [18]. The study of Golan et al. also showed that IFN-β inhibits Th17 cells differentiation [20]. Probably this is one of the mechanisms of action of IFN-β in the treatment of MS [20].

#### 4.2.3. TGF-β Serum Levels

In our study, the higher level of TGF-β after vitamin D supplementation was ob-served. The same effect was reported by Mosayebi and Åivo [15,31]. Both of them sup-posed that the immunoregulatory effect of TGF-β improves the MRI outcomes in RRMS [15].

#### 4.2.4. IFN-γ Serum Levels

In our work, an increase of IFN-γ concentration was observed. Calcitriol has been shown to inhibit in vitro T-cell proliferation and production of IFN-γ [31]. Boonstra et al. reported that vitamin D affects Th cells polarization by inhibiting Th1 (IFN-γ production) and augmenting Th2 cell development [35]. Moreover, their findings suggest that vitamin D acts directly on Th cells and can, in the absence of APC, enhance the development of Th2. This result suggests the beneficial effects of vitamin D in autoimmune disease via prevention of strong Th1 responses (decreasing IFN-γ production). Our results seem to be consistent with the hypothesis of the immunoprotective role of IFN-γ.

### 4.3. Safety of Vitamin D Supplementation. Calcium-Phosphate Metabolism

The proposed daily doses of vitamin D appeared to be safe. A significant decrease in PTH level was found, which was associated with the change in the total 25(OH)D levels. However, the total calcium levels did not change and levels of inorganic phosphorous only slightly increased. Currently, due to the presence of receptors for PTH on the surface of T and B lymphocytes, the potential immunomodulatory role of PTH is being considered [20]. According to Stein, there is a disequilibrium of the PTH-FGF 23–vitamin D axis among patients with RRMS [36]. It means that there is suppression of the parathyroid glands by inappropriately high plasma concentrations of iFGF23 [36]. As a consequence, lower plasma PTH and lower serum 1,25(OH)2D3 to 25(OH)D ratio in MS patients were found [35]. It is important to determine whether plasma PTH and plasma iFGF23 could be used as biomarkers to estimate risk for RRMS and predict disease course [36]. In a more classical approach, it is known that the patients with RRMS have a higher risk of developing osteoporosis and pathological fractures compared to healthy people [2]. They are advised to take vitamin D supplementation both as a prevention of osteopenia and MS progression.

### 4.4. Limitations to the Study

Initially, 83 patients were selected to participate in the study but a substantial number of patients were either excluded or discontinued, which resulted in a significant reduction in the power of analysis. Therefore, it was not possible to perform meaningful analysis of relationships of vitamin D changes with cytokine levels.

Clinical and radiological data of MS course were not analyzed; therefore, we cannot conclude on the possible impact of immunological changes on MS course. It seems that a 12-month observational period is too short to access possible MS progress (in RCTs, the minimum observation period is 2 years), but it is sufficient to observe changes in laboratory results.

RRMS patients treated with different DMT were included in the study, thus their immunological response to vitamin D supplementation may be different. Although, there are numerous publications in which this such relation was not observed [20,29,30]. We decided to not exclude any type of DMT, because the immunomodulatory mode of action of vitamin D is perceived to be independent of any type of DMT. Vitamin D is known to act pleiotropic and none of DMTs modulates VDR receptor. Moreover, patients were re-quired to be on stable dose of DMT for at least 6 months; thus, any new immunological changes were considered to be related to changes in vitamin D levels.

There is no control group in our study. We could not include healthy controls as this does not represent a proper control group. In similar works [30,31], the control group consisted of patients with MS. In our study it was not possible to create a control group with MS patients, because our EC did not agree to administer placebo to MS patients with overt vitamin D deficiency, as beneficial effects of vitamin D supplementation were already proven in this group.

## 5. Conclusions

The results of this study may indicate that long-term supplementation with low doses of vitamin D is sufficient to compensate its deficiency in patients with RRMS and may help to maintain beneficial anti-inflammatory cytokine profile. Moreover, low-dose vitamin D supplementation does not substantially alter calcium-phosphate metabolism. Studies regarding vitamin D deficiency compensation among MS patients should be continued to avoid its overuse.

## Figures and Tables

**Table 1:** Vitamin D supplementation algorithm [28].

25(OH)D Serum Level	Range [nmol/L]	Oral Vitamin D Dose [IU/Daily]
Normal level	≥75–200	Without supplementation
Insufficiency	50–<75	500
Deficiency	25–<50	1000
Severe deficiency	0–<25	1000

**Table 2:** Baseline demographic and clinical characteristics of studied group.

Characteristics	Results (N = 44)
Men/Women [n, %]	11 (25%)/33 (75%)
Age [years, mean ± SD]	38.4 ± 10.1
Age at first symptoms [years, mean ± SD]	28.0 ± 6.2
Age at diagnosis of MS [years, mean ± SD]	32.0 ± 8.3
Disease duration [years, mean ± SD]	5.6 ± 3.4
Polysymptomatic onset [n, %]	24 (55%)
Monosymptomatic onset [n, %]	20 (45%)
EDSS [median, min–max]	1.5 (0–4)
High outdoor physical activity [n, %]	9 (20%)
High consumption of food rich in vitamin D [n, %]	20 (45%)
Smoking [n, %]	10 (23%)
MS Disease Modifying Treatment [n, %]: interferon-beta glatiramer acetate fingolimod ocrelizumab natalizumab	29 (66%) 6 (14%) 4 (9%) 4 (9%) 1 (2%)

EDSS—Expanded Disability Status Scale, SD—Standard Deviation, IQR— interquartile range, MS—Multiple sclerosis.

**Table 3:** Numbers and proportions of patients categorized by the 25(OH)D serum level at baseline and after 12 months of Vitamin D supplementation.

	After 12 Months of Vitamin D Supplementation			
Grouping Based on Serum 25(OH)D Levels	Deficiency (25–<50 nmol/L)	Insufficiency (50–<75 nmol/L)	Normal Level (≥75 nmol/L)	Total n (%) at Baseline
**Baseline values**				
Severe deficiency (0–<25 nmol/L)	1 (2.3%)	4 (9.1%)	6 (13.6%)	11 (25.0%)
Deficiency (25–<50 nmol/L)		3 (6.8%)	17 (38.6%)	20 (45.5%)
Insufficiency (50–<75 nmol/L)		2 (4.5%)	11 (25.0%)	13 (29.5%)
**Total n (%) after 12 months**	1 (2.3%)	9 (20.4%)	34 (77.3%)	44 (100%)

Note: Values are numbers and proportions of patients in the selected groups before and after 12 months participation in the study. Changes assessed with McNemar test for symmetry, *p* < 0.001.

**Table 4:** Median values of serum levels of 25(OH)D, cytokines and calcium-phosphate metabolism parameters at baseline and after 12 months of Vitamin D supplementation.

Parameter	Baseline N = 44	After 12 Months N = 44	Change	*P* *
**25(OH)D [nmol/L]**	39.80 (7.84,73.39)	91.20 (40.99,217.34)	55.36 (–6.12,161.28)	<0.001
**Cytokines [pg/mL]**				
IFN-γ hs	2.14 (1.45,37.30)	3.24 (2.62,43.71)	1.17 (–30.69,41.87)	<0.001
IL-17	5.81 (2.27,10.63)	5.00 (2.06,12.02)	–0.63 (–7.81,8.94)	0.268
IL-10	12.44 (4.88,33.12)	16.76 (6.97,39.24)	2.40 (–25.87,31.17)	0.001
TGF-β	72.30 (40.04,162.58)	100.47 (66.53,172.83)	30.02 (–75.56,105.01)	<0.001
**Calcium-Phosphate Metabolism**				
Total calcium [mmol/L]	2.44 (2.10,2.61)	2.45 (2.33,2.68)	0.01 (–0.17,0.28)	0.118
Inorganic phosphorus [mg/dL]	3.2 (1.9,4.8)	3.5 (2.2,5.1)	0.35 (–1.2,1.6)	0.003
PTH [pg/mL]	46.78 (9.74,97.81)	36.90 (4.42,96.09)	–10.06 (–53.37,41.03)	0.001

Note: Values are median with minimum and maximum in brackets, * Wilcoxon’s test.

**Table 5:** Summary of selected vitamin D supplementation studies in patients with MS.

Authors	Group Size, Vitamin D Dosing [IU/day], Study Duration	Immunomodulatory Results
Sotirchos et al. [18]	N = 40, patients with MS, 10,400 vs. 800 IU, 6 months	The proportion of IL-17-producing CD41^+^ T cells was reduced with higher doses (10,400 IU), but not lower doses (800 IU)
Golan et al. [20]	N = 42, MS patients treated with interferon beta, 800 vs. 4370 IU, 3 months for immunological assessment	IL-17 levels significantly increased in the low dose group, while patients receiving high doses had a heterogeneous IL-17 response
Ashtari et al. [30]	N = 89, MS patients treated with interferon beta, 44 patients on 50,000 IU every 5 days and 45 on placebo, 3 months	IL-10 levels increased significantly after receiving high-dose vitamin D for 3 months
Mosayebi et al. [31]	N = 62, MS patients, 31 pts on 300,000 IU/month i.m., 31 pts on placebo, 6 months	Levels of IL-10 and TGF-β in the vitamin D treatment group were significantly higher than the control group. Unchanged levels of IFN-γ
